# Contaminant-Tolerant Conversion of Polyethylene Waste to α‑Olefins

**DOI:** 10.1021/acssuschemeng.5c10550

**Published:** 2026-01-01

**Authors:** Carlos Posada, Hongwei Sun, Adrian DiMarco, Eric Nuwayo Munyaneza, Oscar Valenzuela, Candace Wall, Guoliang Liu

**Affiliations:** † Department of Chemistry, 1757 Virginia Tech, Blacksburg, Virginia 24061, United States; ‡ Department of Chemical Engineering, Virginia Tech, Blacksburg, Virginia 24061, United States; § Department of Materials Science and Engineering, Virginia Tech, Blacksburg, Virginia 24061, United States; ∥ Macromolecules Innovation Institute, Virginia Tech, Blacksburg, Virginia 24061, United States; ⊥ Division of Nanoscience, Academy of Integrated Science, Virginia Tech, Blacksburg, Virginia 24061, United States

**Keywords:** polyethylene, temperature gradient thermolysis, chemical upcycling, α-olefins, plastic recycling

## Abstract

Polyethylene (PE) is the most widely used polymer in the world. Existing advanced recycling methods are energy-intensive and produce complex products that require further separation and purification. In catalytic chemical upcycling, catalysts are often susceptible to poisoning due to the presence of additives and contaminants in waste streams. Herein, we demonstrate that temperature gradient thermolysis (TGT), a catalyst-free process, can tolerate complex waste streams containing various fillers, additives, and other contaminants. Via TGT, real-life PE waste from complex waste streams selectively produces high-value oil rich in olefins, and ∼90% of these olefins are α-olefins. Additives, fillers, dirt, and biological residues, commonly seen in plastic waste, are absent in the olefin-rich oil. This work highlights the remarkable potential of TGT as a robust approach to produce high-quality α-olefins for subsequent upcycling into high-value chemicals and materials, advancing sustainability.

## Graphical abstract

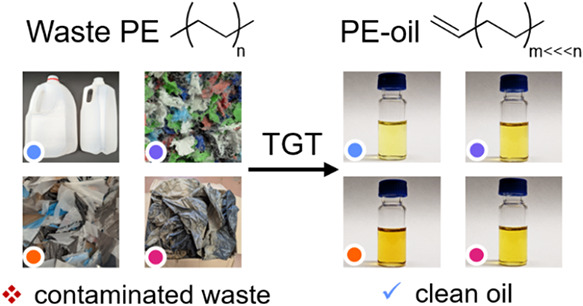

## Introduction

Polyethylene (PE) is the largest-volume plastic produced and used worldwide (∼110 million metric tons (MMT) annually).^1–3^ The reported recycling rate, however, remains low (∼9–11%).^4,5^ Mechanical recycling can potentially divert PE waste from landfills, incineration, or release into the environment,^6^ but the approach requires extensive sorting and cleaning. Plastic waste containing various fillers, additives, and other contaminants (e.g., dyes), especially those in film formats, is commonly rejected by mechanical recyclers.^3,7^ Moreover, the recycled products are typically of lower economic value than the virgin material^8^ due to deteriorated qualities.^9,10^ Thus, there is a strong demand for innovation to treat highly contaminated end-of-life (EOL) waste.

Chemical recycling and upcycling, in principle, are suitable for recalcitrant EOL plastic waste,^11,12^ producing monomers,^13^ syngas,^14^ fuels,^15–18^ and other high-value products such as adhesives^19,20^ and surfactants.^21–23^ Catalytic processes are powerful in converting plastics into desired products with high selectivity,^24–27^ but due to the considerable amount of contaminants and additives, catalyst poisoning is a concern that must be addressed,^13,28–32^ especially for scalable plastic recycling and upcycling. Moreover, despite advances in catalyst design,^33,34^ high temperatures and inert environments at high pressures are still commonly required.^35^ Alternatively, pyrolysis, a noncatalytic thermal process, can tolerate contaminated waste, and yet it requires substantial energy and produces low-quality pyrolysis oil, which necessitates additional refining to yield chemicals suitable for downstream applications.

Recently, we have invented a temperature gradient thermolysis (TGT) process,^22^ which can convert polyolefins into oil and wax products in the absence of any additional chemical reagents, including catalysts, hydrogen, and solvents. Importantly, compared to pyrolysis and high-temperature catalysis that run at temperatures >550 °C or use H_2_ pressures >15 atm, TGT operates at much milder temperatures of <400 °C under atmospheric pressure.

Moreover, the oil and wax products contain substantial amounts of α-olefins with well-controlled chain lengths, which can be upcycled into high-value surfactants.^22,23^ To prepare for future industrial deployment, herein, we evaluate the efficacy of TGT for treating real-life waste streams with various contaminants and additives (e.g., biological residues, inorganic fillers, dyes, and other additives) and assess the quality of the oil products.

## Experimental Section

### Materials

Virgin PE (Formolene) pellets were provided by Procter and Gamble and used as received. Waste PE milk jugs were collected in Blacksburg, VA, rinsed with water, air-dried, and cut into small chunks so that they could be loaded into the reactor through a 24/40 sized joint. Waste PE containers for the House Mix feedstock were collected in Blacksburg, VA, rinsed with water, and air-dried. Labels were removed using mild heat. The plastics were cut into small chunks so that they could be loaded into a coffee blender for grinding and blending purposes. The chunks were blended for 5 min and collected for reaction. PE Films were collected in Blacksburg, VA, and cut into small pieces. PE mulch was donated by Virginia Tech’s Hampton Roads Agricultural Research and Extension Center (AREC) and was lightly washed with deionized water (DI) under mild agitation, air-dried, and cut into smaller chunks prior to use. Nitrogen gas (Nitrogen 99.999% UHP) was purchased from Linde Gas and used as received.

### Instrumentation

GC-MS of oils was performed using a Shimadzu GC2010 Plus equipped with a GCMS-QP2010 SE. Helium was used as the carrier gas, and separations were performed using a DB-5 column (30 m, 250 μm I.D., and a film thickness of 0.25 μm). Operating conditions are outlined in Table 1.

**Table 1:** Description of Parameters Used For GC-MS of Oils

injection port temp.	280 °C
split valve	1/20
purge flow	3 mL/min
constant flow	4.4 mL/min
injection volume	1 μL
column oven initial temp.	50 °C
column initial time	5 min
column oven ramp rate	10 °C/min
column oven final time	280 °C/min
mass spec. transfer line temp.	250 °C
mass spec. database	NIST
MS scan mod range	10–800 amu

GC-MS of gases was performed using an Agilent Technologies 7890A GC system equipped with a 5975C VL MSD detector. Helium was used as the carrier gas and separations were performed using a CP-PoraBOND Q capillary column (Agilent cat. No. 7347; fused silica; length x diameter: 10.0m x 0.25 mm; 0.35 mm outside diameter; 3 μm film thickness). Operating conditions are outlined below in Table 2.

**Table 2:** Description of Parameters Used for GC-MS of Gases

injection port temp.	250 °C
split valve	1/10
purge flow	3 mL/min
total flow	14 mL/min
injection volume	1 μL
column oven initial temp.	35 °C
initial temp. hold	4 min
column initial time	0 min
column oven 2nd temp.	200 °C
column oven ramp rate	10 °C/min
column oven final time	290 °C
column oven final ramp rate	15 °C/min
total run time	26.5 min
mass spec. transfer line temp.	200 °C
interface temperature	250 °C
mass spec. database	Wiley
MS scan mod range	30–550 amu

The oven temperature program was as follows: 35 °C and held for 4 min; ramped to 200 °C at 10 °C/min; then at 15 °C/min to 290 °C: total run time, 26.5 min. GC conditions: column oven temperature, 35 °C; injector temperature, 250 °C; injection mode, split with a split ratio 10:1, 10 mL/min; carrier gas, helium at 6.77 psi; total flow, 14.0 mL/min; septum purge flow, 3.00 mL/min; gas saver flow, 20 mL/min, enabled after 2 min. MS conditions: ion source temperature, 200 °C; interface temperature, 250 °C; mass scan range, 30–550 amu.

### NMR

All ^1^H NMR experiments were performed at 298 K on an Agilent U4-DD2 400 MHz with 32 scans. All spectra were recorded using 10 mg of thermolysis oil in deuterated chloroform.

### TGA

All thermogravimetric analysis was performed using a TA Instruments Discovery TGA 5500 under constant nitrogen flow (40 mL/min) and ramp rate of 10 °C/min to a final temperature of 700 °C.

### FTIR

All Fourier transform infrared spectroscopy was performed using an Agilent Technologies Cary 630 FTIR. Scans were performed in the range of 650 to 4000 cm^–1^ and were collected using 128 scans at a resolution of 4 cm^–1^. Prior to the collection of each spectrum, 32 background scans were performed. Happ Genzel apodization and Mertz phase correction were used for all samples.

### Microwave Digestion and ICP-MS

Feedstock and oil microwave digestions were performed using ∼0.25 and 0.10 g of material, respectively. Samples were charged to separate Teflon digestion vessels, and 10 mL of 70% nitric acid was added and gently swirled (for 15 min). The vessels were sealed and put into a microwave digestion system (Anton Parr Multiwave GO Plus). Vessels were heated up to 200 °C in 15 min and held at 200 °C for 15 min. After microwave digestion, the vessels were cooled to ambient temperature and opened for collection. The solutions were diluted 50 times with deionized water and analyzed using a Thermo Electron iCAP RQ ICPMS.

### Raman Spectroscopy

Raman spectroscopy was performed on a Horiba XploRA Plus Confocal Raman Microscope using a resolution of 1200 gr/mm and a minimum of 6 acquisitions at 8 s each. The laser intensity was kept at 1–25% total power and varied depending on the precursor plastic. Samples were prepared by suspending the residual material left after thermolysis in chloroform and drop casting the resultant suspension onto a glass slide. Enough sample was drop-cast to prevent background signal from the glass substrate.

### TGT Reactor Setup

Temperature gradient thermolysis (TGT) reactors were constructed utilizing common glassware to generate three temperature zones according to Figure 1. A Glas-Col heating mantle was used to heat the reaction flask (temperature zone one – *T*_1_) and was calibrated to the desired temperature utilizing a laser thermometer prior to reaction. Temperature zone two (*T*_2_) was achieved using another heater and the temperature was calibrated with a K-Type temperature probe. Temperature zone 3 (*T*_3_) was achieved using a constant flow of room temperature water through a reflux condenser. To maintain an inert environment, the reflux condenser was capped with a rubber septum and affixed with a balloon. Glassware joints were sealed using Teflon sleeve joint adapters. Temperatures were optimized according to Table S1.

**Figure 1:**
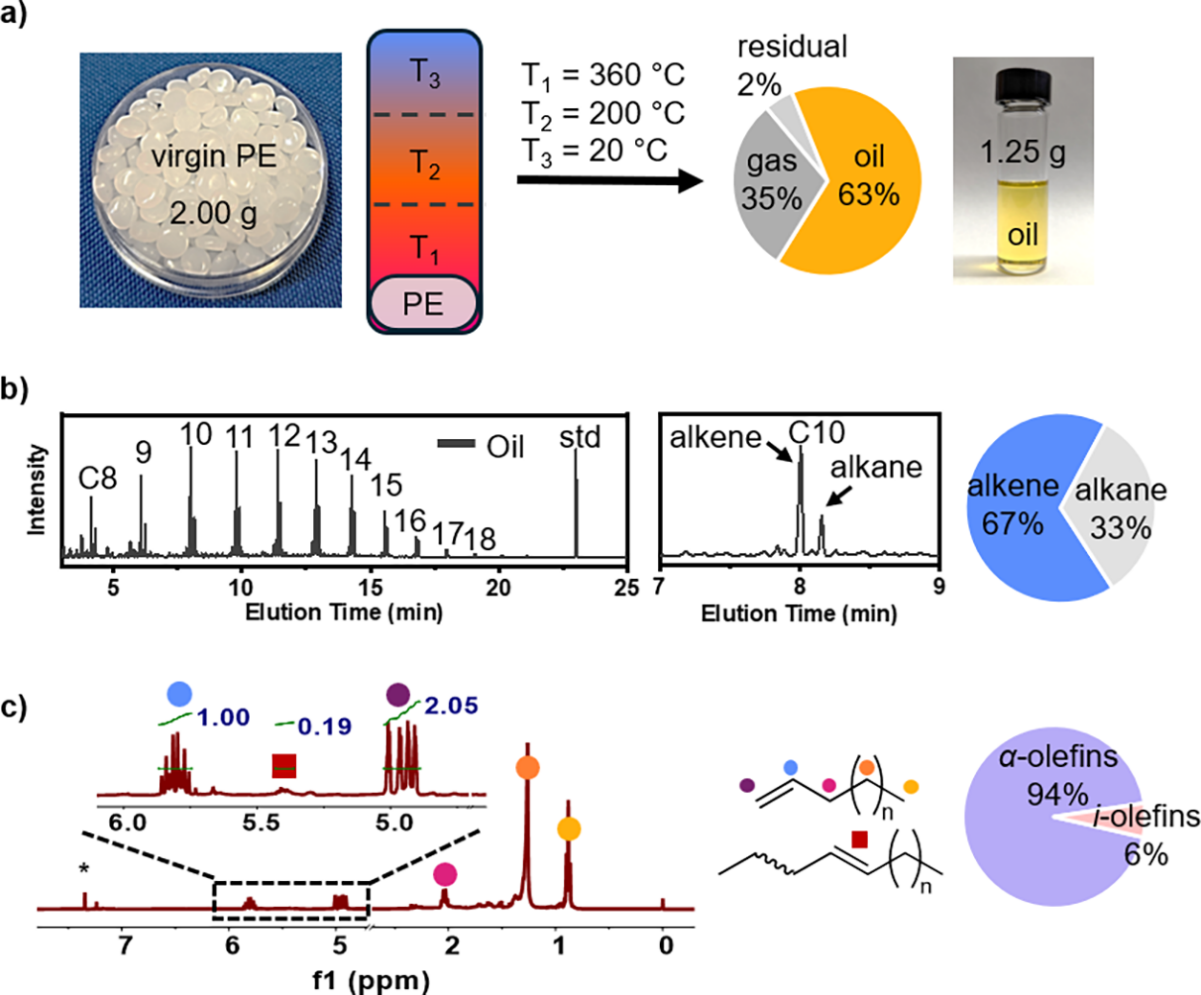
Temperature gradient thermolysis of virgin HDPE pellets with (a) *T*_1_ as the deconstruction zone, *T*_2_ as the condensation zone, and *T*_3_ as the product collection zone. The reaction produces oil (∼63%), gas (∼35%), and solid residue (∼2%). (b) GC-MS analysis of the oil (67% alkenes and 33% alkane) using a linear C_22_H_46_ standard. The zoom-in view highlights the C10 range, showing mostly alkene. (c) ^1^H NMR analysis of the thermolysis oil shows α-olefins (94%) and internal olefins (i-olefins, 6%).

### TGT of PE Waste

PE waste (typically 2 g) was charged into a premassed 100 mL round-bottom flask. After loading with heat transfer media, the flask was affixed to the TGT reactor. The reactor was purged with N_2_ for 15 min and sealed with an internal pressure of ∼1 atm. Temperature zones 2 and 3 were established at 200 and 25 °C, respectively, for 15 min prior to the initiation of thermolysis. PE waste was heated at 340, 360, or 380 °C for 2 h. Heating was ceased, and the reaction was cooled naturally to room temperature prior to sample collection.

### Oil Collection

Following completion of TGT, the oil fraction was drained from *T*_3_ using a stopcock at the bottom of the collection zone. Samples were added to a premassed vial, and the mass of oil was determined according to eq 1, below.

1 $${\mathrm{Mass}}_{\mathrm{oil}}={\mathrm{Mass}}_{\mathrm{oil}+\mathrm{vial}}-{\mathrm{Mass}}_{\mathrm{vial}}$$

### Gas Collection

Gas samples were collected via a rubber balloon that was purged with N_2_ and affixed to the end of the T_3_ zone for the duration of the thermolysis reaction. Gas from the balloon was purged through a clean GC-vial that had been prepurged with N_2_. The vial was sealed using parafilm on a cap containing a noncut rubber septum. Gas samples were then manually injected and characterized utilizing the GC-MS conditions outlined above.

### Analyses of Thermolysis Products

The thermolysis oil products were collected and massed with a Metler Toledo XSE105 DualRange analytical balance. The mass of residual products was calculated by massing the T_1_ reaction flask on a Metler Toledo XSE105 DualRange analytical balance and subtracting the mass of the flask prior to the reaction. The yields of the oil (wt %_oil_) and residues (wt %_Residue_) were calculated by dividing the masses of the oil and residues by the mass of the PE waste loaded into the reactor, respectively. Due to the difficulty to directly weight the mass of the gas phase products, the wt % yields of gas phase products were determined according to eq 2

2 $${\mathrm{wt}\,\;\%}_{\mathrm{Gas}}=100\%-\left({\mathrm{wt}\,\;\%}_{\mathrm{Oil}}+{\mathrm{wt}\,\;\%}_{\mathrm{Residue}}\right)$$

The thermolysis oil products were further analyzed using gas chromatography–mass spectroscopy (GC-MS). All chromatograms were integrated using the Origin Peak Analyzer function. The integration ratios of olefin and paraffin peaks were compared. To determine the ratio of α and internal olefins, peak integrations from ^1^H NMR were used according to eq 3

3 $$\left(\frac{\int H_{\mathrm{terminal}\;\mathrm{alkenes}}}{\int H_{\mathrm{all}\;a\mathrm{lkenes}}}\right)\times 100$$

where H_terminal alkenes_ was the integration value for protons corresponding to terminal alkenes (∼4.95 and 5.80 ppm) and H_all alkenes_ was the integration value for protons corresponding to internal alkenes (∼5.40 ppm) (Figure S1).

## Results and Discussion

Before evaluating the capability of TGT to produce α-olefins from real-world waste PE, we first established a baseline for our process using commercial HDPE pellets. Similar to our previous reports,^22,23^ our thermolysis reactor contained a temperature gradient, which featured a hot zone to initiate the chemical deconstruction of plastics and a cold zone to quench the reaction and minimize gas production. Through extensive reaction condition screening, we have modified our temperature profiles with three temperature zones of *T*_1_ = 360 °C, *T*_2_ = 200 °C, and *T*_3_ = 20 °C (Figure 1, Table S1). Under this condition, the thermolysis of PE produced oil as the predominant product (∼63 wt %), followed by gas (∼35 wt %), and residual solids (∼2 wt %). GC-MS analysis of the thermolysis oil showed alkenes and alkanes mostly in the range of C8–C15, with minor peaks of C16–C18 and an alkene-to-alkane ratio of ∼2:1. ^1^H NMR analysis revealed high selectivity for α-olefins (∼94%).

Linear α-olefins were the dominant products from HDPE deconstruction, and chain length distribution was well controlled. In contrast to constant-temperature pyrolysis, TGT produced negligible amounts of BTX (benzene, toluene, and xylenes)^36–38^ and internal alkenes.^16,39^ A residual chloroform peak and a trace benzene peak were also observed at 7.2 and 7.3 ppm, respectively.

Strikingly, our TGT process showed substantially higher yields of α-olefins than alternative methods in the literature.^16,39^ The high α-olefin selectivity likely resulted from judicious control over the temperature gradient. The relatively high *T*_2_ ensures more oligomers in the vapor phase, avoiding premature condensation and minimizing subsequent recombination. The low *T*_3_ ensures full condensation of the target products and, due to insufficient energy, prevents olefin saturation reactions to produce alkanes. Thus, our TGT process is effective in converting PE to α-olefin-rich oils.

Encouraged by these results, we further tested the applicability of our TGT process to several waste PE feedstocks from a variety of end-use applications and with diverse coloration and form-factor. Milk jugs represented high-quality HDPE of food grade, while house mix featured colored bottles, tubes, and other containers utilized for cosmetics, detergents, and food packaging. PE films, such as grocery bags, which are often rejected by mechanical recyclers due to potential machinery clogging, contained various levels of fillers (e.g., CaCO_3_, Na_2_SO_4_, SiO_2_).^16,40^ As an ultimate challenge with potential bioresidues and other agricultural contaminants (Figure S7), plastic mulch was collected from a local outdoor strawberry farm after six months of field service.

Each waste stream was subjected to the same thermolysis conditions as virgin PE and produced a significant quantity of oil (Figure 2). The milk jugs and house mix feedstocks showed the highest oil yields of ∼64 and 62 wt %, respectively. PE films and plastic mulch offered lower oil yields of ∼45 and 53 wt %, respectively. The thermolysis oils exhibited varying hues of yellow, depending on the type of feedstock, but all oils showed no visible particulates or microplastics. Integration of the alkene peaks in the ^1^H NMR spectra (Figure S1) revealed ∼90% selectivity toward terminal olefins instead of internal olefins, and the selectivity decreased in the order of milk jugs > house mix > PE films > plastic mulch. All wastes produced ∼33–37 wt % gas, but the solid residue yield ranged from ∼3 to 21 wt %.

**Figure 2:**
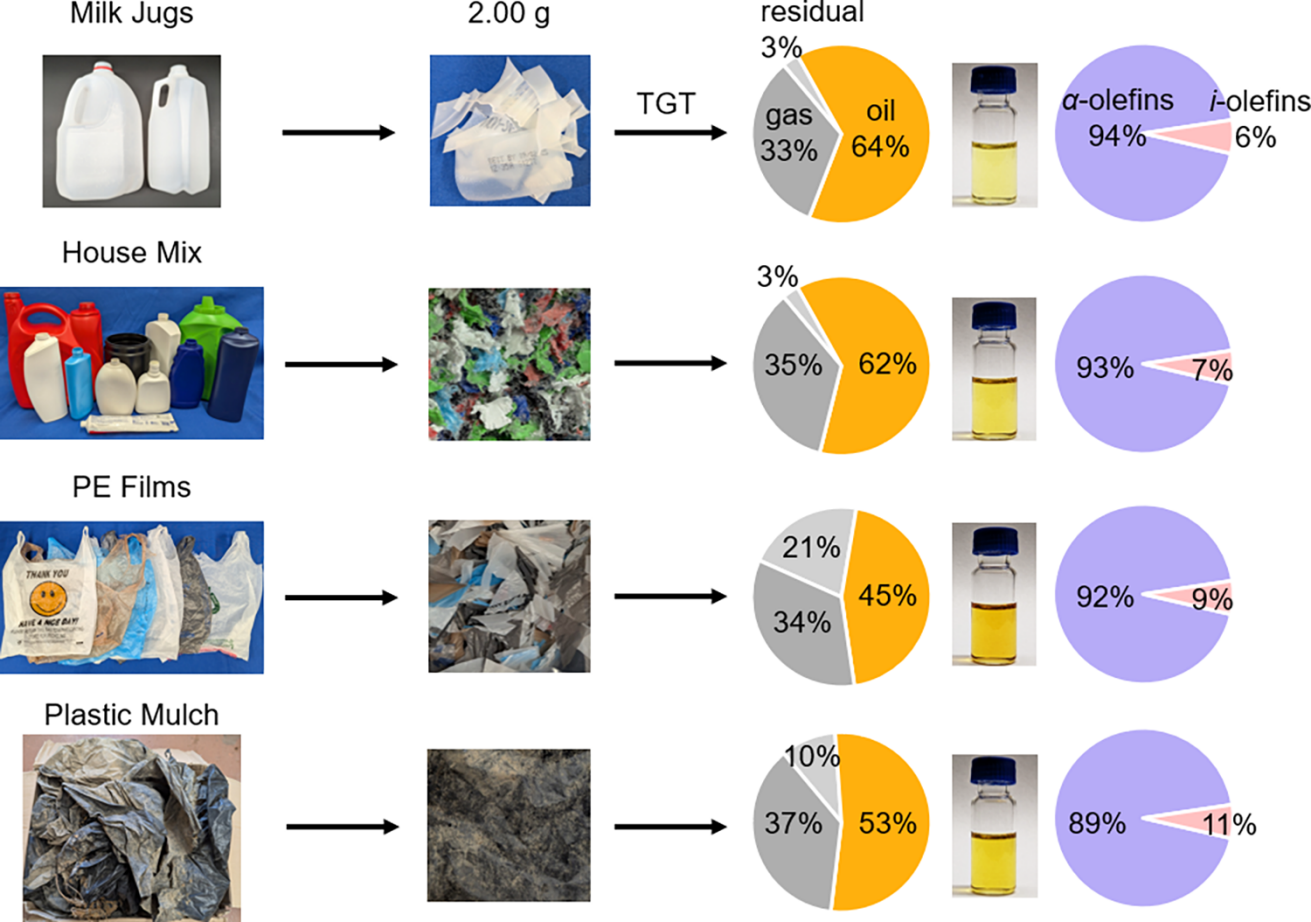
Temperature gradient thermolysis of milk jugs, house mix containers, PE films, and plastic mulch, along with the corresponding product phases and α-olefin fractions in the oil.

Among all the tested waste feedstocks, milk jugs yielded products that were most similar to virgin PE pellets. We rationalize this outcome because plastic formulations for food packaging are of higher purity and contain fewer additives due to Food and Drug Administration (FDA) requirements. As a result, the oil yields, coloration, and selectivity of milk jugs and virgin PE were comparable. In contrast, a variety of organic additives, inorganic fillers, and dyes are well reported for use in PE containers, PE films, and plastic mulch. Residual biomass was also observed in the plastic mulch (Figure S7). All these additives, fillers, and contaminants likely caused the deviated product yields and thermolysis oil coloration.

To further understand how the additives and contaminants altered the thermolysis product composition, we first assessed the thermal and chemical profiles of all tested feedstocks (Figure 3). Thermogravimetric analysis (TGA) showed that the virgin HDPE and milk jugs had the highest decomposition temperature at 5 wt % loss (*T*_d5%_) of ∼460 °C. The house mix began to decompose at a slightly lower temperature (*T*_d5%_ = ∼450 °C). The PE films and plastic mulch showed the lowest *T*_d5%_ of ∼370 and 435 °C, respectively, likely due to the additives such as plasticizers and slip agents used during film processing.^41,42^ Additionally, virgin HDPE and milk jugs were completely decomposed after 500 °C. At 700 °C, house mix, PE films, and plastic mulch showed char yields of ∼2, 14, and 12%, respectively, which explained the various oil yields from these waste streams. Interestingly, PE films exhibited an additional weight loss at ∼600 °C, attributed to decomposition of CaCO_3_ commonly used in the plastic bag manufacturing process.^40,43^

**Figure 3:**
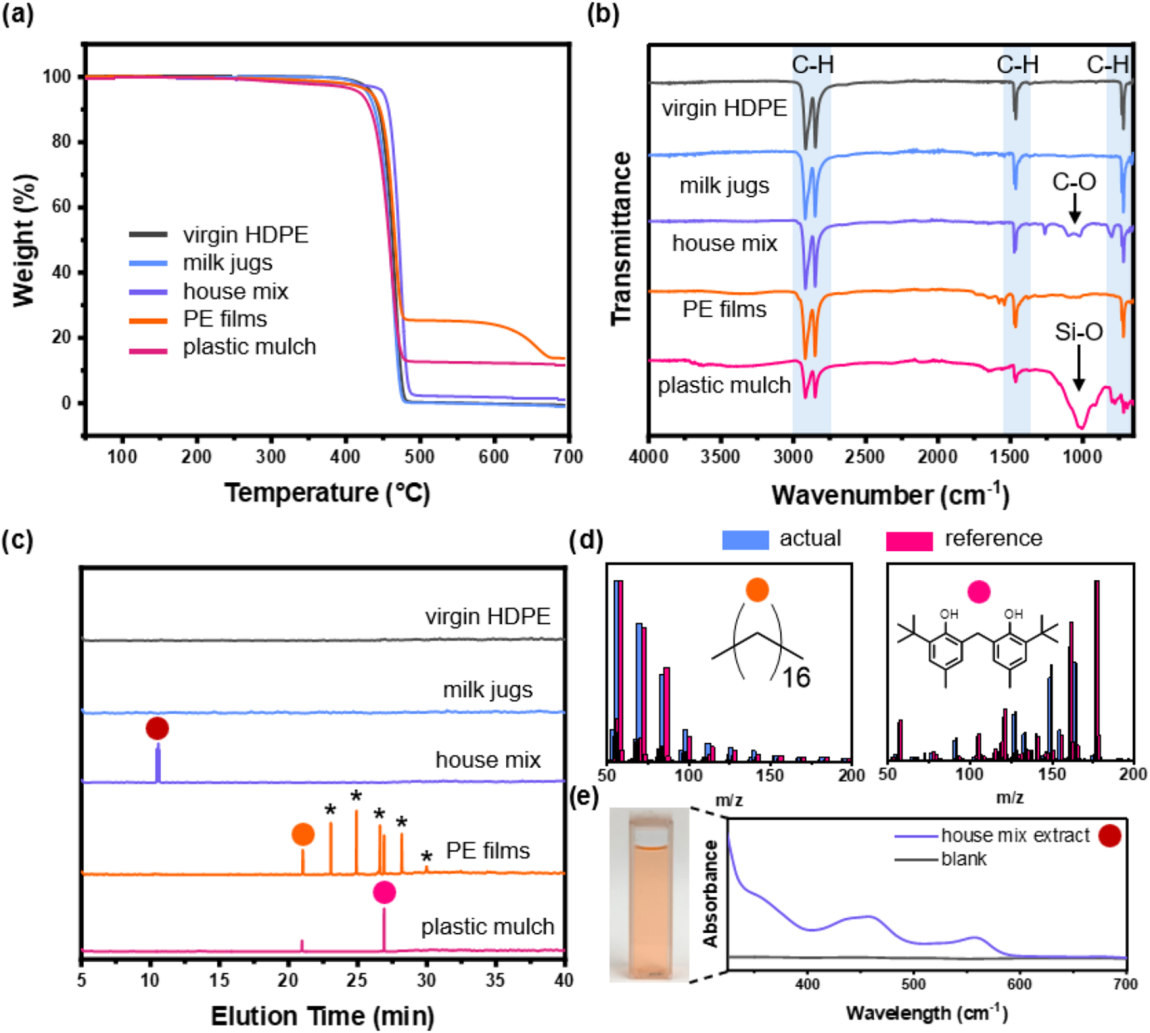
Characterization of the plastic feedstocks using (a) TGA and (b) FTIR. (c) GC and (d) mass spectra of the compounds extracted from the plastic feedstocks. (e) UV–vis spectrum of house mix extract.

The chemical compositions of the plastic waste were probed by FTIR analysis, showing significant alkane C–H stretching and bending at 800, 1480, and 2800 cm^–1^ in all tested feedstocks (Figure 3b). Except for virgin HDPE and milk jugs, house mix and PE films displayed an additional peak at 1000 cm^–1^, likely corresponding to C–O stretching in plasticizers, antioxidants, and dyes.^44^ Plastic mulch exhibited a significantly broader Si–O stretch, attributed to soil residues.

Soxhlet extraction of organic residues and subsequent analysis via GC-MS and UV–vis spectroscopy were leveraged to reveal the composition of organic residues in each plastic feedstock (Figures 3c,d, S8, and S9). No additives were detected in the virgin HDPE; however, several chemical additives were present in the other waste. GC analysis of the Soxhlet extract from house mix exhibited peaks at ∼10 min, and the MS analyses of these compounds showed low matches to any compounds in the NIST database. However, the extract exhibited a red-ish color and was analyzed using UV–vis spectroscopy. The absorbance peaks at ∼350, 450, and 550 cm^–1^ indicate the extracts were likely dye molecules, but the molecular structures could not be determined. PE films revealed a series of elution peaks at ∼21–30 min. Mass spectroscopy suggested that these peaks were C18–C23 paraffins (Figure S9), which are commonly used in PE films for plasticization purposes.^45^ The plastic mulch also showed a C18 peak, which was probably added during the manufacturing process.^44^ Additionally, both PE films and plastic mulch showed a peak at ∼27 min, which matched a diphenolic molecule commonly used as an antioxidant.^46^

While all feedstocks exhibited similar thermal stability compared to virgin PE pellets, differences in char yield likely indicate the presence and quantity of inorganic fillers. For example, the mass loss observed in the PE films feedstock at ∼600 °C likely corresponds to the decomposition of CaCO_3_ present within the material. Additionally, SiO_2_ likely contributes to a significant portion of the remaining mass because it is thermally stable under the tested conditions (max temperature, 700 °C). Finally, plastic mulch also contained residual dirt from the field where it was used and might also contain carbon black to impart UV resistance. The minimal char yield in the virgin PE, milk jug, and house mix indicates the absence of these materials within the tested samples. The results corroborate the observed product compositions in Figure 2, insofar as residual yields match the observed char yields (Table S2). Thus, we concluded that inorganic fillers directly accounted for the increased residual yields observed from the TGT of PE films and plastic mulch.

FTIR of the waste and GC-MS analysis of Soxhlet extracts provided evidence for the presence of both polar and nonpolar organic additives in the waste feedstocks. Phenolic antioxidants, commonly included in PE formulations, were observed in the PE films and plastic mulch.^46^ We identified well-reported slip agents such as paraffinic waxes. Finally, the series of peaks observed in the house mix likely corresponded to organic dyes responsible for the strong coloration exhibited by the bottles and tubes (Figure 2).

To understand how organic additives influenced the oil products, we compared waste-derived oils to oil made from virgin PE (Figures S2–S6). A series of aliphatic alkane and alkene peaks appeared in all GC-MS traces, corresponding to C9 to C20 (Figure 4a). Comparing the integration of alkene and alkane peaks indicated that the alkene fraction was highest in the PE films oil (67%) and the lowest in the house mix and plastic mulch oils (62%). The chromatograms of all tested oils exhibited small quantities of xylene and ethylbenzene (elution time, ∼6 min). The oils for PE films and plastic mulch exhibited a barely noticeable peak at ∼24.5 min (likely pyrene, ∼90% match). No other significant contaminants were observed.

**Figure 4:**
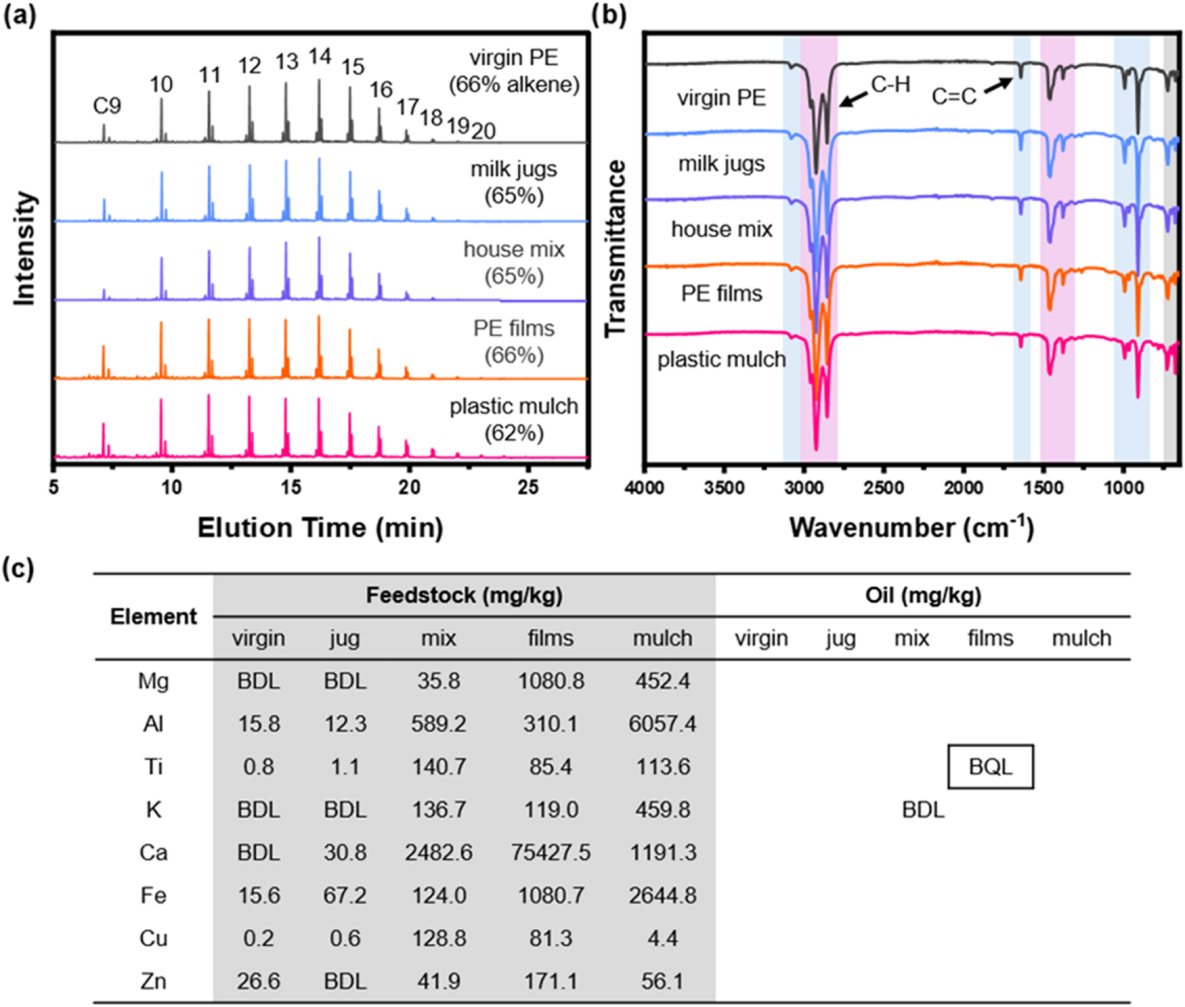
Analysis of TGT oils using (a) GC and (b) FTIR of the thermolysis oils from various plastic feedstocks. Light blue and pink stripes highlight signals from alkenes and alkanes, respectively. The light gray stripe highlights signals that could arise from either alkenes or alkanes. (c) ICP-MS analysis of the metal elements in the plastic feedstocks and the thermolysis oils. Metal concentrations in all oils were either below the detection limit (BDL) or below the quantitation limit (BQL).

FTIR spectra of the thermolysis oils showed alkene C–H stretching at 3080 cm^–1^ as well as a medium intensity alkene C=C stretching at 1645 cm^–1^. A prominent vinyl alkene signal appeared at 900 cm^–1^, along with a peak at 990 cm^–1^ that may result from both alkene (C=C) and vinylic proton (=C–H) bending. Peaks at 675 and 730 cm^–1^ could be attributed to bending modes of either alkenes or alkanes. Strong C–H stretching bands were present at 2860, 2920, and 2960 cm^–1^, accompanied by a C–H bending peak at 1460 cm^–1^. An alkane bending feature was also noted at 1380 cm^–1^. No absorbance bands corresponding to C–O, C=O, or −OH functionalities were detected (Figure 4b).

We compared the metal content of feedstocks and their respective thermolysis oils (Figure 4c). Most plastic waste feedstocks contained a substantial amount of metal content. The house mix, PE films, and plastic mulch contained higher concentrations of metals than virgin PE and milk jugs. Magnesium and potassium concentrations were below detection limits in both virgin PE and milk jugs, and calcium was also below detection limits in virgin PE. Despite the high concentrations of metal contents in the plastic waste, the derived concentrations (eq S1) of most metals in all TGT oils were below detection limits (BDL) (Tables S3 and S4, respectively), except for titanium, which was below the quantitation limit (BQL).

Chemical analysis of TGT oils revealed that all waste-derived oils exhibited high purity, even when produced from feedstocks containing organic additives and substantial amounts of metals such as Ca in PE films. Despite the presence of antioxidants, biological residues, dyes, and metals, additional significant peaks were not observed in the GC-MS traces for the house mix, PE films, and plastic mulch feedstocks. FTIR analysis corroborated these results, insofar as house mix, PE films, and plastic mulch thermolysis oils exhibited similar compositions to virgin PE and milk jugs. As a result, both FTIR and GC-MS analyses of thermolysis oils suggested that the discoloration observed in nonfood-grade waste plastics likely arose from the trace inclusion of polyaromatic hydrocarbons (PAH). ^1^H NMR spectra of TGT oils (Figure S1) corroborated these findings, and ICP-MS analyses of both feedstocks and oils showed that metal contamination was negligible.

After investigating the influence of organic additives, contaminants, and metals on the quality of oils produced from waste PE thermolysis, our attention shifted to analyzing gaseous and residual products (Figure 5). Similar to thermolysis oils, the gaseous products contained both alkenes and alkanes of C2–C6. The peak at ∼1.4 min was the most prominent in all samples and matched propylene (>90% match). The high selectivity to propylene and low concentrations of C2 suggested that it was difficult to induce further chain scission from C3 to C2. The MS data of C5–C6 products showed a relatively low match with n-pentene and n-hexene, indicating potential branching or cyclization of C5 and C6 products to produce isomers. The peak at ∼0.1 min was attributed to adventitious carbon dioxide. No additional major peaks were observed, suggesting that heavier additives and contaminants were effectively filtered by the utilized TGT process.

**Figure 5:**
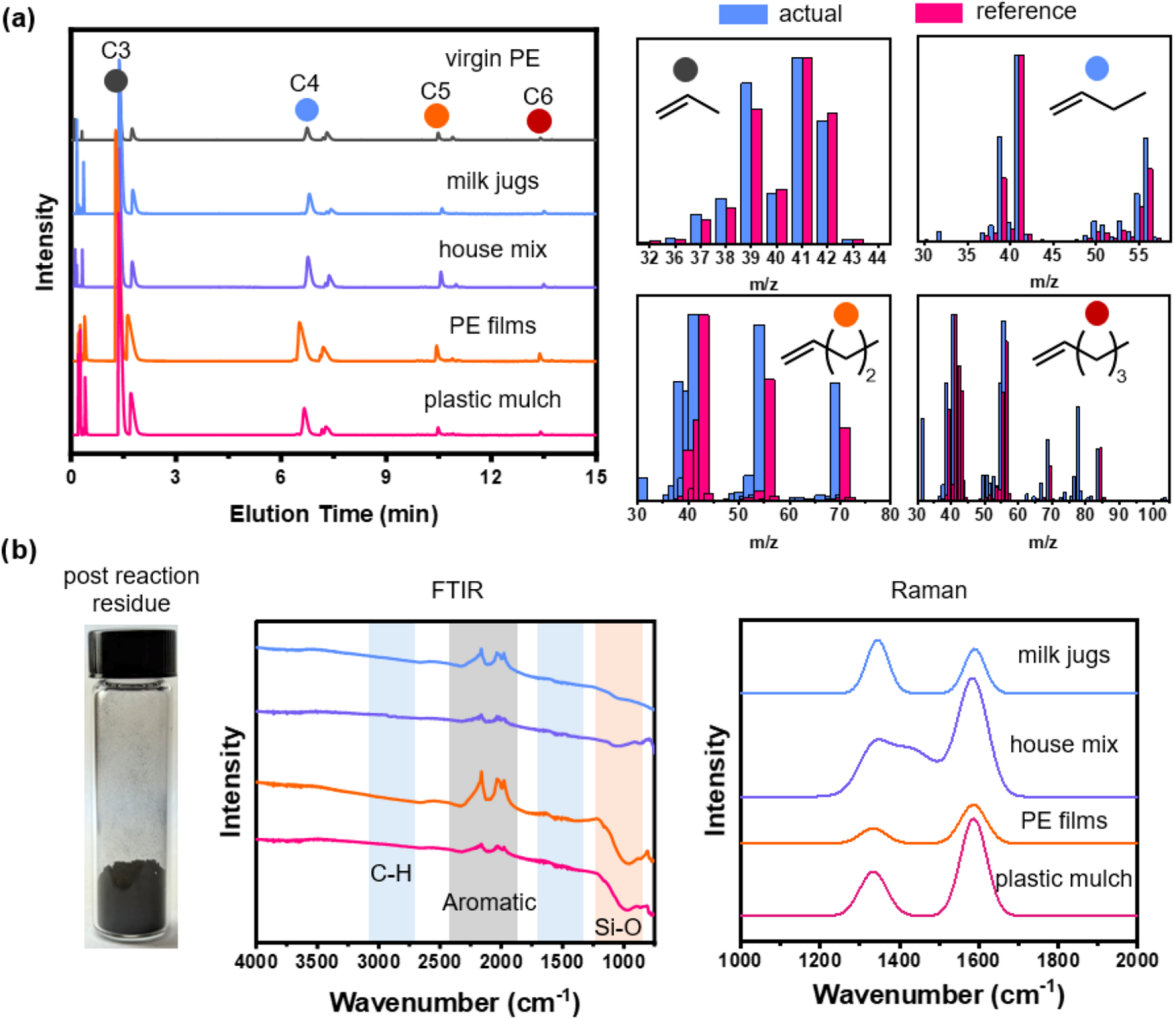
Analysis of TGT byproducts with (a) GC-MS of the gaseous products show C2–C6 hydrocarbons. (b) FTIR and Raman spectroscopy of the solid residue show mostly carbonaceous products.

FTIR analysis of the residual solid revealed the presence of aromatic moieties, with stretches at ∼1925–2320 cm^–1^. Si–O stretching was also observed at ∼1000 cm^–1^ in the PE films and plastic mulch residues, thus inorganic fillers like SiO_2_ were confirmed in the residual char after thermolysis. No other significant stretching signals were observed. The graphitic degree of residual solid was studied using Raman spectroscopy by comparing the disordered (D) and graphitic (G) peaks at ∼1340 and 1590 cm^–1^, respectively. Milk jugs exhibited the highest D:G ratio (1.2), and PE films the lowest (0.4). House mix and plastic mulch showed D:G ratios of 0.8 and 0.5, respectively. Thus, we determined that solid residues were mostly carbonaceous materials^47,48^ from degraded PE, dyes, and low-volatility antioxidants. Inorganic fillers were detected in the residue if present in the original feedstocks. The lack of metal content in all TGT oils suggested that these metals remained in the residue because there were no mechanisms for them to volatilize under the tested conditions.

To probe the crystallinity of the residual chars, we determined the full-width-half-max (FWHM) values for the D and G peaks. Except for house mix, all other solid residues displayed FWHM values of ∼80 cm^–1^ for both the D and G peaks. The G peak of the house mix residue showed a FWHM of 95 cm^–1^, which indicated increased defects in the material. We were unable to determine the FWHM of the D peak for the house mix because a shoulder peak obscured the baseline, which may have resulted from the degradation of dyes with conjugated cyclic backbones. Considering that the solid residues from plastic thermolysis remain relatively unexplored, future investigations may study the particle size, porosity, absorptivity, and char yields to determine their suitability for carbon black production.

After determining that temperature gradient thermolysis exhibits a high degree of tolerance for additives and contaminants within feedstocks, we evaluated the effect of further increasing the reaction scale (Figure 6). Thermolysis of 20 g of virgin HDPE yielded 11.5 g of oil (58 wt % yield), 7.4 g of residue (37 wt % yield), and 1.1 g of gas (5.0 wt % yield). GC-MS analysis of the thermolysis oil showed alkenes and alkanes in the range of C9–C20. Peak integration of the chromatogram revealed an alkene yield of 44.1% and an alkane yield of 55.9%. The α-olefin yield was determined via ^1^H NMR with a trimethoxybenzene internal standard and found to be 86.0 mol %.

**Figure 6:**
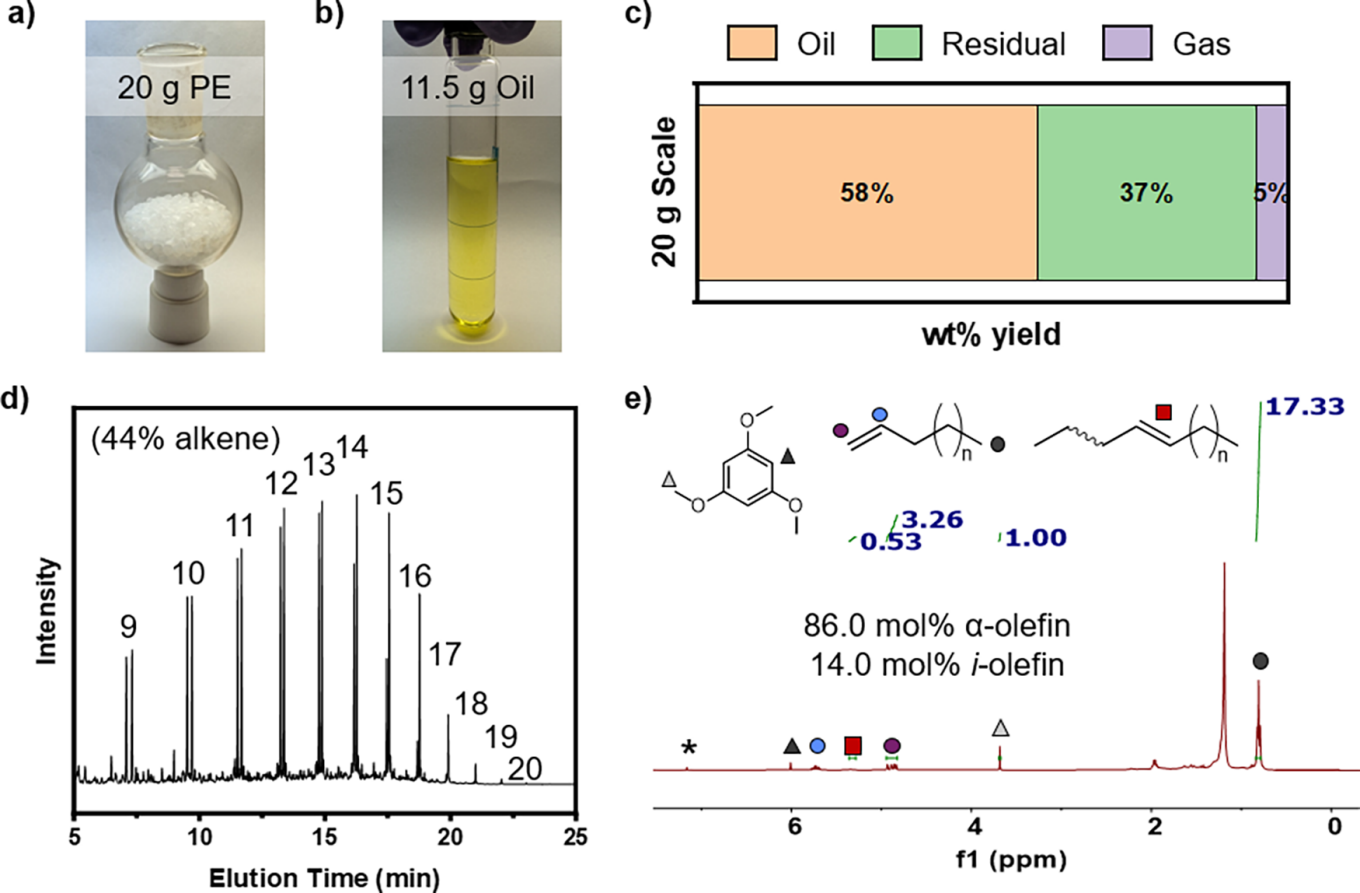
Large scale thermolysis reaction of (a) 20 g of virgin polyethylene to primarily produce (b) oil as well as (c) residual and gaseous products. (d) GC-MS analysis of the oil shows 44.1% alkenes and 55.9% alkanes. (e) ^1^H NMR analysis of the thermolysis oil shows 86.0 mol % α-olefins.

In the scaled-up thermolysis of PE, 95.6% of olefins were within the desirable range of C9 to C15. Additionally, linear α-olefins were the major products in the oil. The selectivity to linear α-olefins was lower in the 20-g reaction than in the 2-g reaction, but it can be optimized in the future. In our current design, it was difficult to uniformly heat and react all polymer chains because of the large amount of PE melt in the reactor. The large-scale reaction produced more reactive intermediates, which might condense and subsequently cross-link in the melt, leading to higher yields of residual products. Future designs of flow reactors could prevent cross-linking in the degradation zone of the reactor, thus improving the oil and α-olefin yields. Regardless, the high selectivity of α-olefins without the use of catalysts highlights the robustness of temperature gradient thermolysis, even after increasing the reaction scale 20-fold from our previous works.

## Conclusions

Herein, we report a method for converting highly contaminated postconsumer PE waste into high-value α-olefins using TGT. The TGT process tolerates various contaminants and additives used in production processes and introduced during customer practices, offering high selectivity toward α-olefin oil. Regardless of the quality of waste feedstocks, the TGT oils exhibited high purity, and the amount of metals commonly seen in the plastic waste streams were not detectable by ICP-MS in the thermolysis oil. The production of high-quality oils from waste feedstocks without the use of any additional chemical reagents, such as catalysts, hydrogen, and solvents, highlights the potential of TGT for scalable and robust processing of real-life waste, paving a foundation for future industrial deployment in plastic recycling facilities.

## Supplementary Material



## Notes

^1^H NMR of α-olefin oils produced from each feedstock (Figure S1); gas chromatograms and the wt % yield of each product in the mixture for all tested feedstocks (Figures S2–S6); photograph of crude plastic mulch feedstock (Figure S7); zoomed in view of the gas chromatograms for compounds in the House Mix Soxhlet extract and the PE films Soxhlet extract (Figures S8 and S9) and mass spectra for compounds in the PE Films Soxhlet extract (Figure S9); thermolysis results while varying temperature zones 1 and 2 (Table 1); comparison of the char yields from TGA and residual solid yields from TGT reactions for each feedstock (Table 2); concentrations of various metals in PE waste feedstocks and thermolysis oils (Table 3); detection and quantitation limits for ICP-MS (Table 4) (PDF)
