# Supplementary material for: Contaminant-Tolerant Conversion of Polyethylene Waste to α‑Olefins
Source: ACS Sustain Chem Eng. 2026 Jan 1;14(2):1057–66. doi: 10.1021/acssuschemeng.5c10550 (PMC12820977; doi:10.1021/acssuschemeng.5c10550)
Supplement: Supplementary file 1 [file sc5c10550_si_001.pdf]

Supplementary Materials

For

Contaminant-tolerant Conversion of Polyethylene Waste to α-Olefins

Carlos Posada†, Hongwei Sun†, Adrian DiMarco‡, Eric Nuwayo Munyaneza†, Oscar Valenzuela†, Candace Wall†, Guoliang Liu†,‡,#,^,$*

†Virginia Tech Department of Chemistry, Virginia Tech, 1040 Drillfield Dr., Blacksburg, VA 24061, USA

‡Department of Chemical Engineering, Virginia Tech, 635 Prices Fork Rd., Blacksburg, VA 24061, USA

#Department of Materials Science and Engineering, Virginia Tech, 445 Old Turner St. #213, Blacksburg, VA 24061, USA

^Macromolecules Innovation Institute, Virginia Tech, 240 West Campus Dr., Blacksburg, VA 24061, USA

$Division of Nanoscience, Academy of Integrated Science, Virginia Tech, 400 Stranger St., Blacksburg, VA 24061, USA

*Corresponding author: Dr. Guoliang (Greg) Liu

E-mail: gliu1@vt.edu

Number of Pages: 12

Number of Figures: 9

Number of Tables: 4

The Supporting Information contains:

Supporting Figures:

Figure S1 ^{1}H NMR of α-olefin oils produced from each feedstock.

Figure S2 Gas chromatogram of Virgin PE Oil and the wt.% yield of each product in mixture.

Figure S3 Gas chromatogram of Milk Jugs Oil and the wt.% yield of each product in mixture.

Figure S4 Gas chromatogram of House Mix Oil and the wt.% yield of each product in mixture.

Figure S5 Gas chromatogram of PE Films Oil and the wt.% yield of each product in mixture.

Figure S6 Gas chromatogram of Plastic Mulch Oil and the wt.% yield of each product in mixture.

Figure S7 Photograph of crude plastic mulch feedstock.

Figure S8 Zoomed in view of the gas chromatogram for compounds in the House Mix Soxhlet extract.

Figure S9 Gas chromatogram and corresponding mass spectra for compounds in the PE Films Soxhlet extract.

Supporting Tables:

Table 1 Thermolysis results while varying temperature zones 1 and 2.

Table 2 Comparison of the char yields from TGA and residual solid yields from TGT reactions for each feedstock.

Table 3 Concentrations of various metals in PE waste feedstocks and thermolysis oils.

Table 4 Detection and quantitation limits for ICP-MS.

Analyses of thermolysis products.

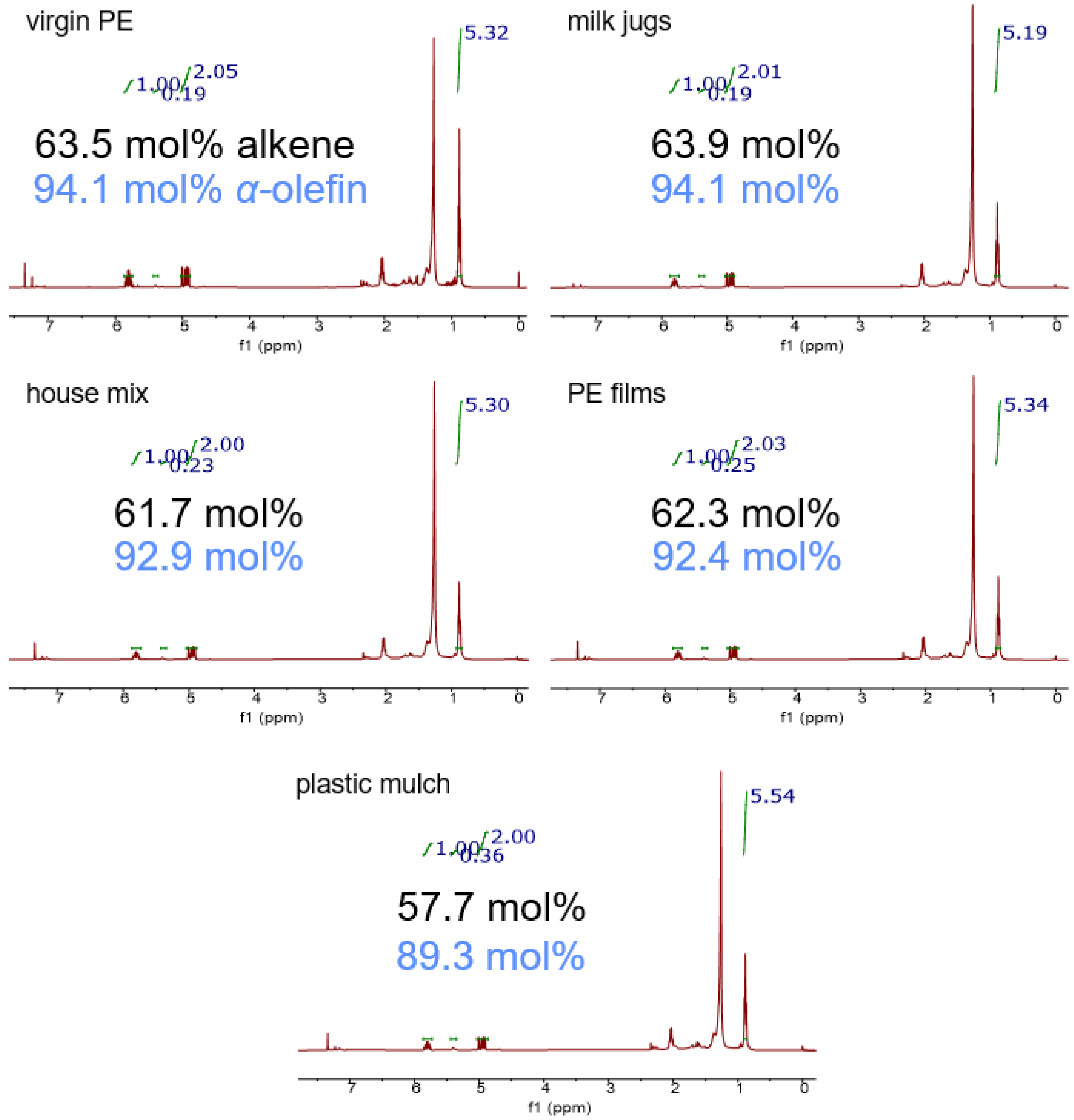

Figure S1. ^{1}H NMR of HDPE derived thermolysis oils showing the mol% alkene yield (in black) and the *α*-olefin yield (in blue) as calculated by comparing the integration values for terminal and internal alkene peaks.

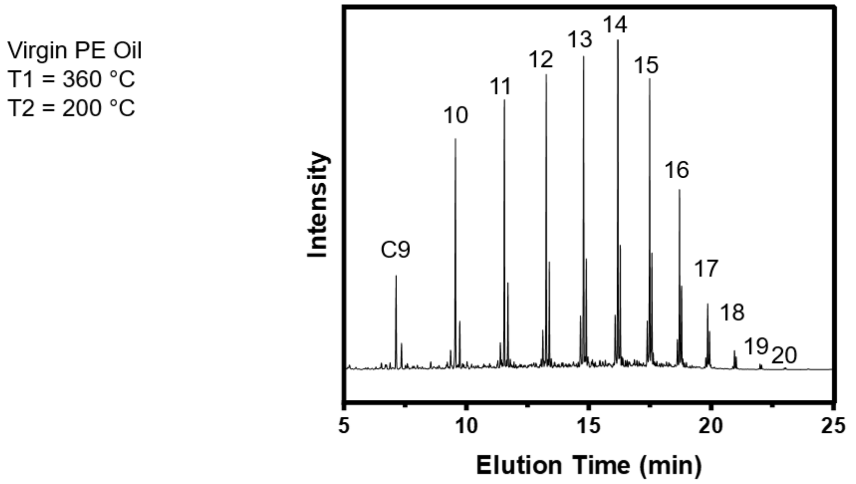

# of Carbon Atoms	C9	C10	C11	C12	C13	C14	C15	C16	C17	C18	> C18	C9-13	Total
Alkene	3.04	7.62	8.32	8.80	9.55	10.53	9.01	5.36	2.00	0.60	0.28	37.33	65.11
Alkane	0.93	1.53	2.51	3.02	3.21	3.58	4.02	2.83	1.17	0.40	0.27	11.20	23.47
Diene	0.27	0.79	0.88	1.49	2.19	2.35	1.74	1.10	0.42	0.13	0.05	5.62	11.41
Yield (%)	4.24	9.94	11.71	13.31	14.95	16.47	14.77	9.30	3.59	1.13	0.60	54.15	100.00

Figure S2. Wt.% yields of different carbon chain lengths and functionalities within virgin PE oil.

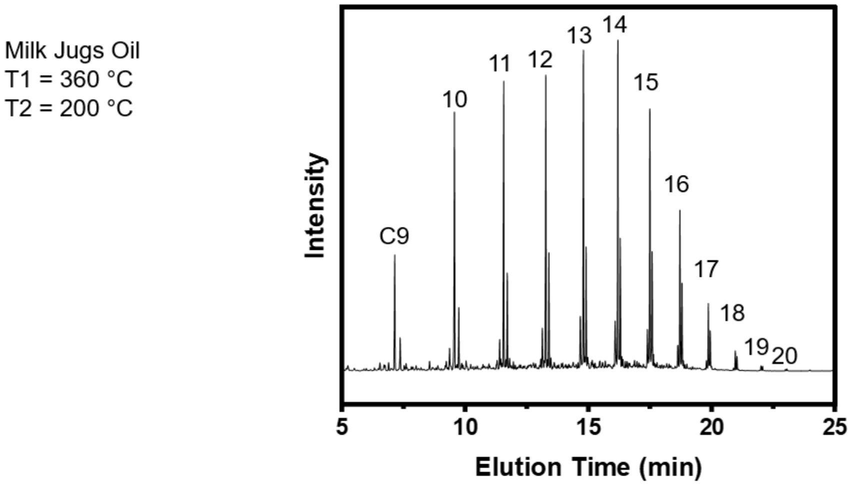

# of Carbon Atoms	C9	C10	C11	C12	C13	C14	C15	C16	C17	C18	> C18	C9-13	Total
Alkene	3.772	8.330	8.836	9.136	9.554	10.022	7.900	4.779	1.853	0.573	0.242	39.628	64.997
Alkane	1.130	1.871	2.793	3.227	3.327	3.707	3.581	2.568	1.164	0.439	0.260	12.35	24.067
Diene	0.310	0.911	0.979	1.528	2.167	2.061	1.527	0.937	0.357	0.118	0.042	5.895	10.937
Yield (%)	5.21	11.11	12.61	13.89	15.05	15.79	13.01	8.28	3.37	1.13	0.55	57.87	100.00

Figure S3. Wt.% yields of different carbon chain lengths and functionalities within milk jugs oil.

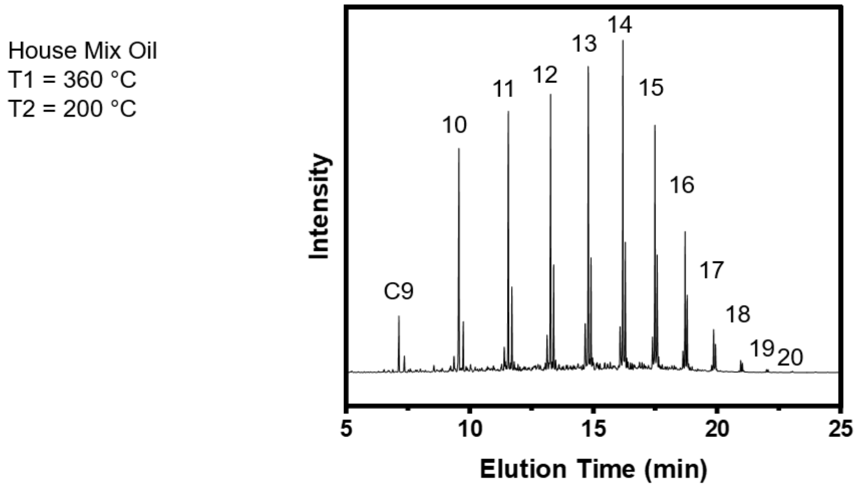

# of Carbon Atoms	C9	C10	C11	C12	C13	C14	C15	C16	C17	C18	> C18	C9-13	Total
Alkene	1.865	7.897	9.035	9.710	10.240	10.710	8.646	4.531	1.446	0.399	0.162	38.747	64.641
Alkane	0.602	1.651	2.763	3.417	3.678	4.046	4.229	2.668	0.930	0.318	0.186	12.111	24.488
Diene	0.130	0.643	0.928	1.559	2.278	2.369	1.666	0.912	0.288	0.074	0.022	5.538	10.869
Yield (%)	2.60	10.19	12.73	14.69	16.20	17.12	14.54	8.11	2.66	0.79	0.37	56.40	100.00

Figure S4. Wt.% yields of different carbon chain lengths and functionalities within house mix oil.

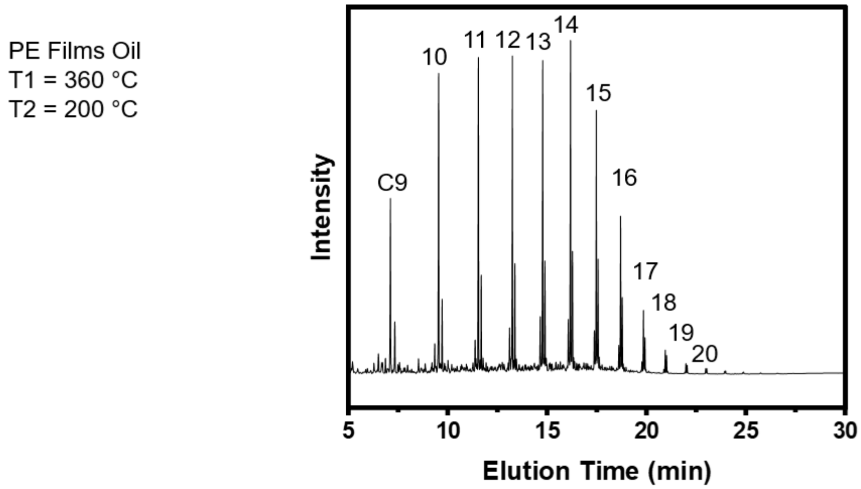

# of Carbon Atoms	C9	C10	C11	C12	C13	C14	C15	C16	C17	C18	> C18	C9-13	Total
Alkene	6.028	9.338	9.039	8.681	8.816	9.178	7.141	4.125	1.661	0.661	0.627	41.902	65.295
Alkane	1.674	2.044	2.702	2.960	3.020	3.282	3.042	2.248	0.998	0.497	0.682	12.400	23.149
Diene	0.546	0.969	1.034	1.635	2.177	2.024	1.496	0.964	0.412	0.169	0.129	6.361	11.555
Yield (%)	8.25	12.35	12.78	13.28	14.01	14.48	11.68	7.34	3.07	1.33	1.44	60.66	100.00

Figure S5. Wt.% yields of different carbon chain lengths and functionalities within PE films oil.

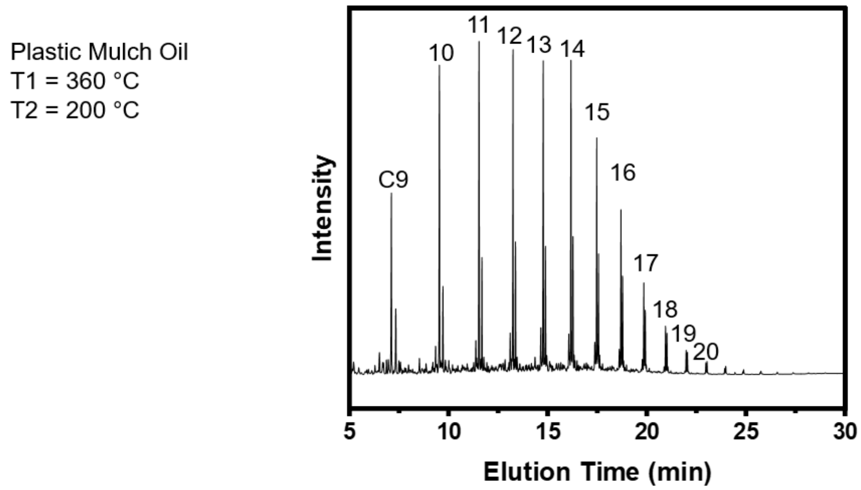

# of Carbon Atoms	C9	C10	C11	C12	C13	C14	C15	C16	C17	C18	> C18	C9-13	Total
Alkene	5.531	8.645	8.582	8.309	7.880	7.699	5.996	4.036	2.309	1.252	1.392	38.947	61.631
Alkane	2.014	2.309	2.969	3.222	3.248	3.366	3.074	2.648	1.621	1.067	1.939	13.762	27.477
Diene	0.538	0.992	0.999	1.517	1.891	1.622	1.328	0.890	0.550	0.289	0.281	5.937	10.897
Yield (%)	8.08	11.95	12.55	13.05	13.02	12.69	10.40	7.57	4.48	2.61	3.60	58.20	100.00

Figure S6. Wt.% yields of different carbon chain lengths and functionalities within plastic mulch oil.

Characterization of PE additive and contaminant.

Prior to reaction, all wastes were visually examined and photographed. Varying degrees of contamination with dirt and biological residues were noted, with plastic mulch exhibiting the most contamination during the usage process (**Figure S7**). Using TGA, the char yields were compared to the wt.% yield of residuals, which was used to estimate the wt.% of nonvolatile inorganic additives and contaminants in the waste(**Table S2**).

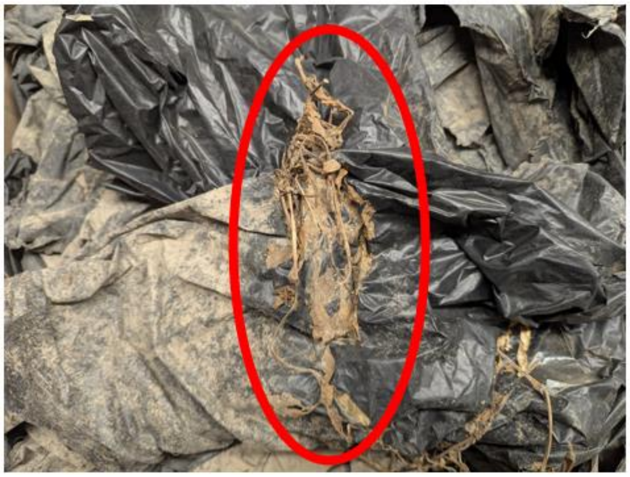

Figure S7. Contaminated film waste (plastic mulch) containing dead plant matter (circled in red) and field sediment.

Organic soluble additives were extracted from plastic waste via Soxhlet extraction using a mixture of cyclohexane and isopropanol (mixing ratio by volume, 1:9). The extracted solution was concentrated using rotary evaporation at 40 °C and 150 mbar. GC-MS analysis revealed a variety of peaks, which were compared to the NIST database (**Figures S8** and **S9)**. ICP-MS analyses were used to detect and quantify the metal content in PE waste and thermolysis oils (**Tables S3 and S4)**.

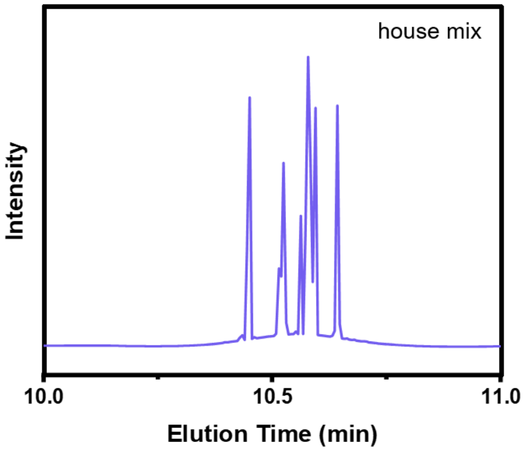

Figure S8. Gas chromatogram of house mix Soxhlet extract. All peaks exhibited a poor match to any known compounds in the NIST mass spectroscopy database.

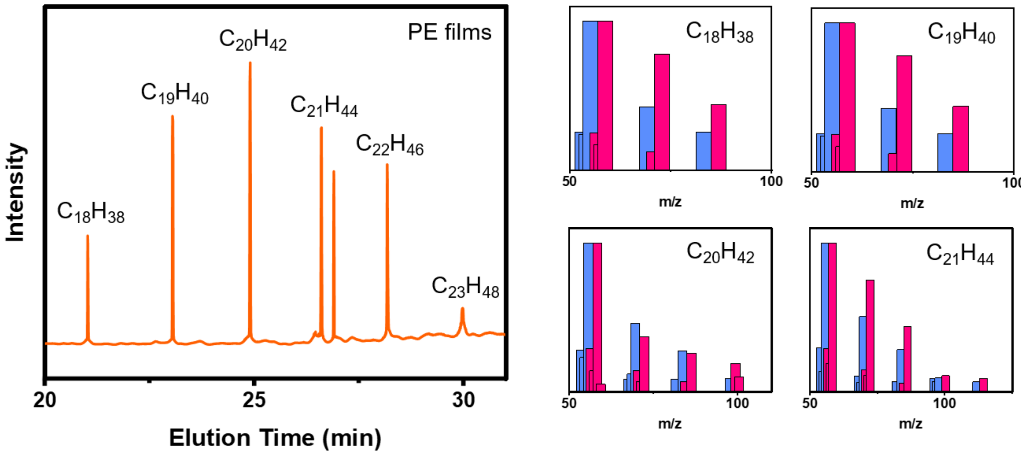

Figure S9. Mass spectroscopy data comparing experimental and NIST reference database values for paraffin peaks in the GC of the PE films Soxhlet extract. Due to issues with maintaining volatilization, peaks at ~ 27.5 and 30 min were unable to be matched; however, their identity was assumed due to the periodic nature of paraffin mixtures.

Table S1. Optimizing thermolysis conditions.

T_1 (°C)	T_2 (°C)	Oil (wt.%)	Gas (wt.%)	Residual (wt.%)	Alkene Yield (wt.%)*
340	200	51.7	44.6	3.7	37.4
360	200	59.4	37.9	2.7	42.5
380	200	56.1	41.4	2.5	41.2
360	160	51.7	40.5	7.8	37.2
360	180	56.1	40.1	3.8	41.2

* The alkene yield is the weight of alkenes in the oil divided by the total mass of PE feedstock.

Table S2. Comparison of the char yield from TGA and residual solid yields from TGT reactions.

Feedstock	Residual wt.%	Char Yield @ 500 °C
virgin PE	2	0
milk jugs	3	0
house mix	3	2
PE films	21	25
plastic mulch	10	12

Table S3. Metal concentration in PE waste feedstocks and thermolysis oils.

Element	Feedstock (ppb)					Oil (ppb)				
	virgin	jug	mix	films	mulch	virgin	jug	mix	films	mulch
Mg	0.6	0.8	18.1	551.4	227.5	0.0	0.0	0.0	0.0	0.0
Al	7.9	6.2	298.2	158.2	3046.9	0.3	0.1	0.2	0.3	0.2
Ti	0.4	0.5	71.2	43.6	57.2	0.0	0.1	0.0	0.5	0.0
K	-2.3	2.0	69.2	60.7	231.3	0.0	0.0	0.0	0.0	0.0
Ca	0.4	15.4	1256.5	38484.6	599.2	0.0	0.0	0.0	0.0	0.0
Fe	7.9	33.6	62.7	551.4	1330.3	0.0	0.0	0.0	0.0	0.0
Cu	0.1	0.3	65.2	41.5	2.2	0.0	0.0	0.0	0.0	0.0
Zn	13.4	0.1	21.2	87.3	28.2	0.0	0.0	0.0	0.0	0.0

The values from **Table S3** were converted from ppb to mg/kg according to equation S1.

$$\left(\frac{[M]\ (ppb) \times \left(\frac{1}{1000}\right)\left({}^{mg}/_{L}\right) \times V_{HNO_3}\ (L) \times (Dilution\ Factor)}{m_{sample}\ (mg)}\right) \times 10^6 \qquad (S1)$$

where [M] is the respective metal concentration within the sample, V_{HNO3} is the volume of nitric acid used (10 mL), the samples were diluted with 50 mL of deionized water (DI), and m_{sample} was the mass of the digested sample. Values below the detection or quantitation limit were listed as BDL or BQL, respectively.

Table S4. ICP-MS detection and quantitation limits.

Element	Detection Limit (ppb)	Quantitation Limit (ppb)
Mg	1.11	5.00
Al	1.45	10.00
Ti	0.3	0.10
K	2.14	10.00
Ca	5.14	50.00
Fe	0.36	5.00
Cu	0.08	1.00
Zn	0.13	1.00
